# Supplementary material for: An In-Depth Study on the Metabolite Profile and Biological Properties of Primula auriculata Extracts: A Fascinating Sparkle on the Way from Nature to Functional Applications
Source: Antioxidants (Basel). 2022 Jul 15;11(7):1377. doi: 10.3390/antiox11071377 (PMC9312287; doi:10.3390/antiox11071377)
Supplement: Supplementary file 1 [file antioxidants-11-01377-s001.zip › antioxidants-1790691-supplementary.pdf]

Article

An in-depth study on the metabolite profile and biological properties of *Primula auriculata* extracts: a fascinating sparkle on the way from nature to functional applications

İnci Kurt-Celep[1], Dimitrina Zheleva-Dimitrova[2], Reneta Gevrenova[2], Abdullahi Ibrahim Uba[3], Gokhan Zengin[4*], Evren Yıldıztugay[5], Carene Marie Nancy Picot-Allain[6], José Manuel Lorenzo[7,8], Mohammad Fawzi Mahomoodally[6], Domenico Montesano[9*]

[1]*Yeditepe University, Faculty of Pharmacy, Department of Pharmacognosy, 34755, Ataşehir, Istanbul, Turkey,* incikurt00@gmail.com (I.K.C)

[2]*Department of Pharmacognosy, Faculty of Pharmacy, Medical University-Sofia, Sofia, Bulgaria,* dimizheleva@gmail.com (D.Z); rgevrenova@gmail.com (R.G)

[3]*Department of Molecular Biology and Genetics, Faculty of Engineering and Natural Sciences, Kadir Has University, Istanbul 34083, Turkey,* abdullahi.iu2@gmail.com (A.I.U)

[4]*Physiology and Biochemistry Research Laboratory, Department of Biology, Science Faculty, Selcuk University, Campus, 42130, Konya, Turkey,* gokhanzengin@selcuk.edu.tr (G.Z)

[5]*Department of Biotechnology, Science Faculty, Selcuk University, Campus, Konya, Turkey,* eytugay@gmail.com (E.Y)

[6]*Department of Health Sciences, Faculty of Medicine and Health Sciences, University of Mauritius, Réduit, Mauritius,* picotcarene@gmail.com (C.M..N.P); f.mahomoodally@uom.ac.mu (M.F.M)

[7]*Centro Tecnológico de la Carne de Galicia, Rúa Galicia Nº 4, Parque Tecnológico de Galicia, San Cibrao das Viñas, 32900 Ourense, Spain,* jmlorenzo@ceteca.net (J.M.L)

[8]*Universidade de Vigo, Área de Tecnoloxía dos Alimentos, Facultade de Ciencias, 32004 Ourense, Spain*

[9]*Department of Pharmacy, University of Naples Federico II, via D. Montesano 49, 80131 Naples, Italy,* domenico.montesano@unina.it (D.M)

* Correspondence: gokhanzengin@selcuk.edu.tr (Dr. Gokhan ZENGIN); domenico.montesano@unina.it (Dr. Domenico Montesano)

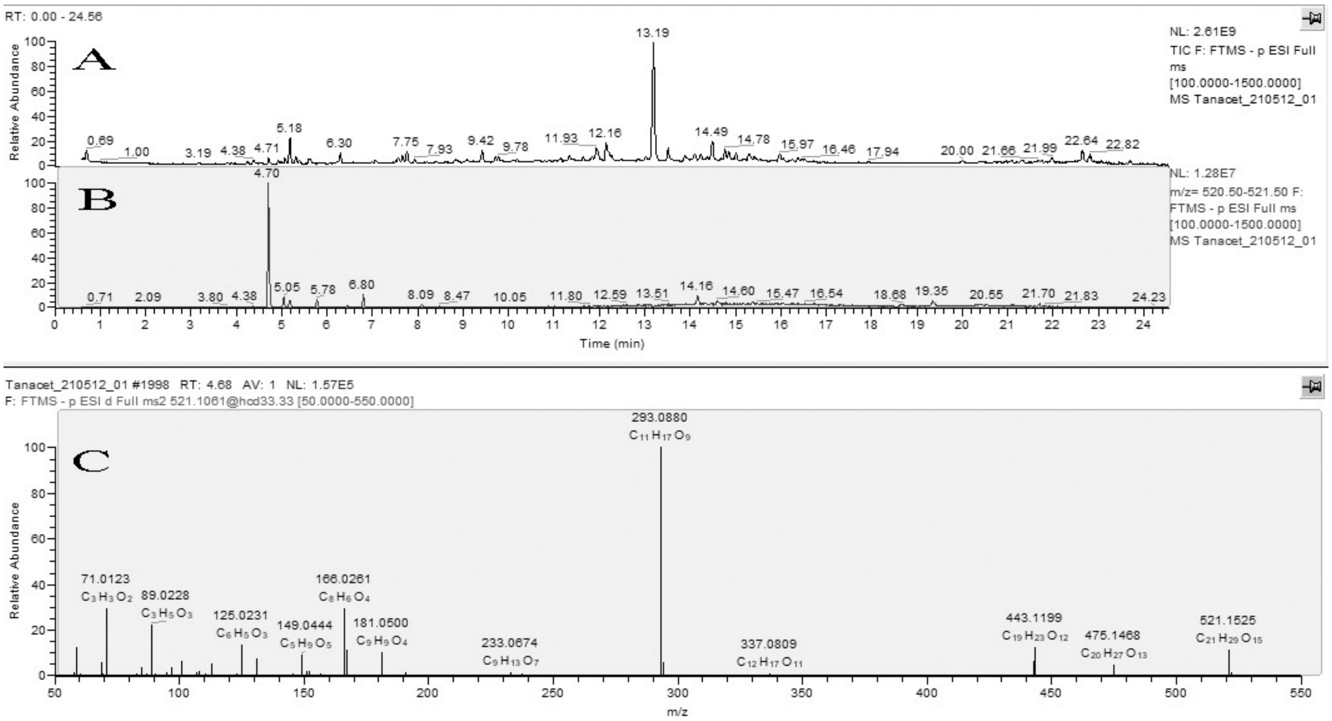

Figure S1. Total ion chromatogram of *P. auriculata* aerial parts ethyl acetate extract in negative ion mode (A), extracted ion chromatogram of compound 5 at *m/z* 475.1468 (B), and MS/MS fragmentation of compound 5 (C)

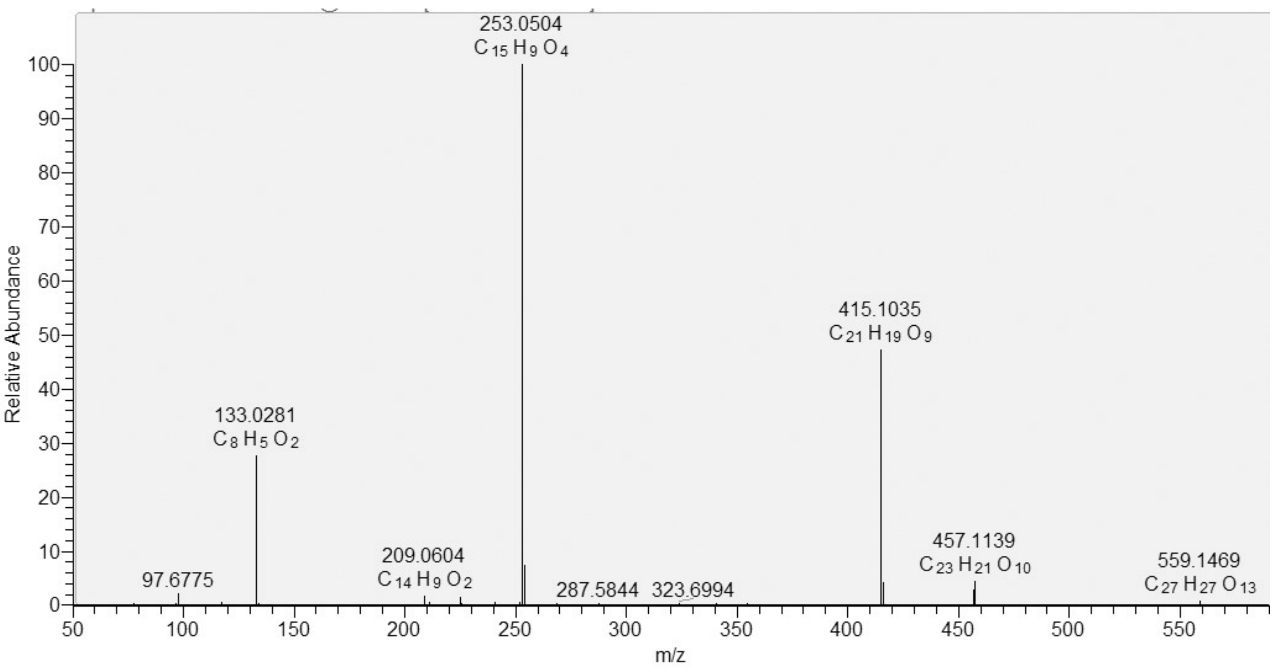

Figure S2. MS/MS spectrum of compound 32 at *m/z* 559.145

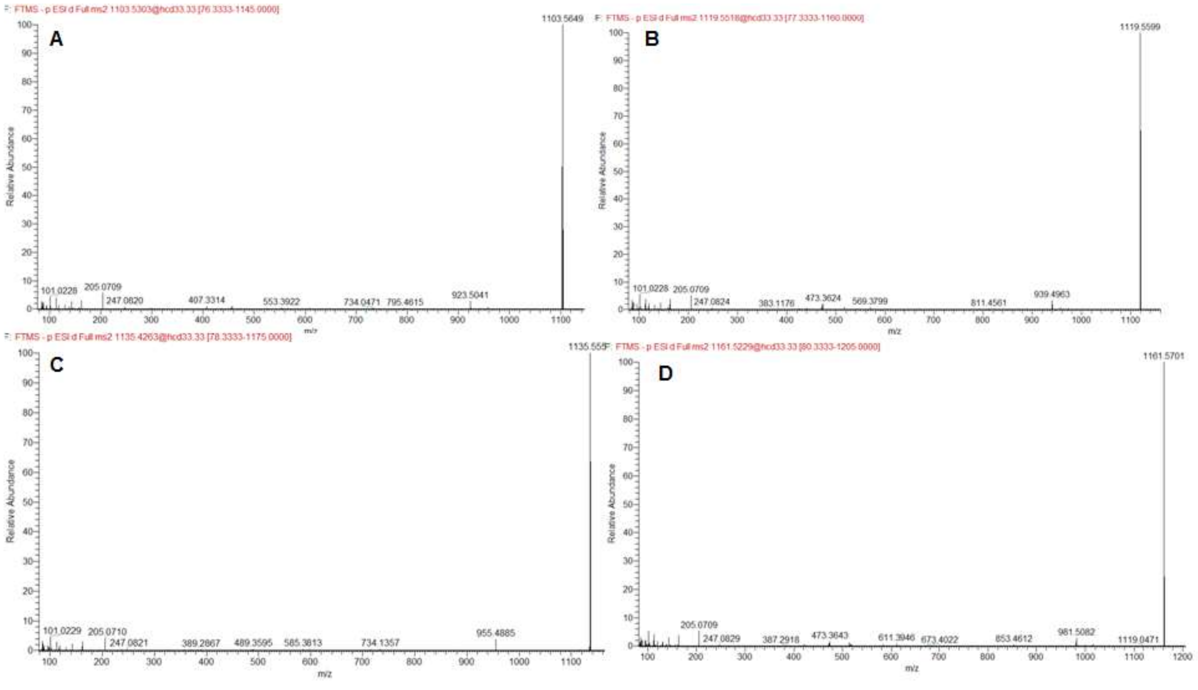

Figure S3. MS/MS spectra of *Primula auriculata* saponins at *m/z* 1103.565 (A); 1119.559 (B); 1135.555 (C); 1161.570 (D).

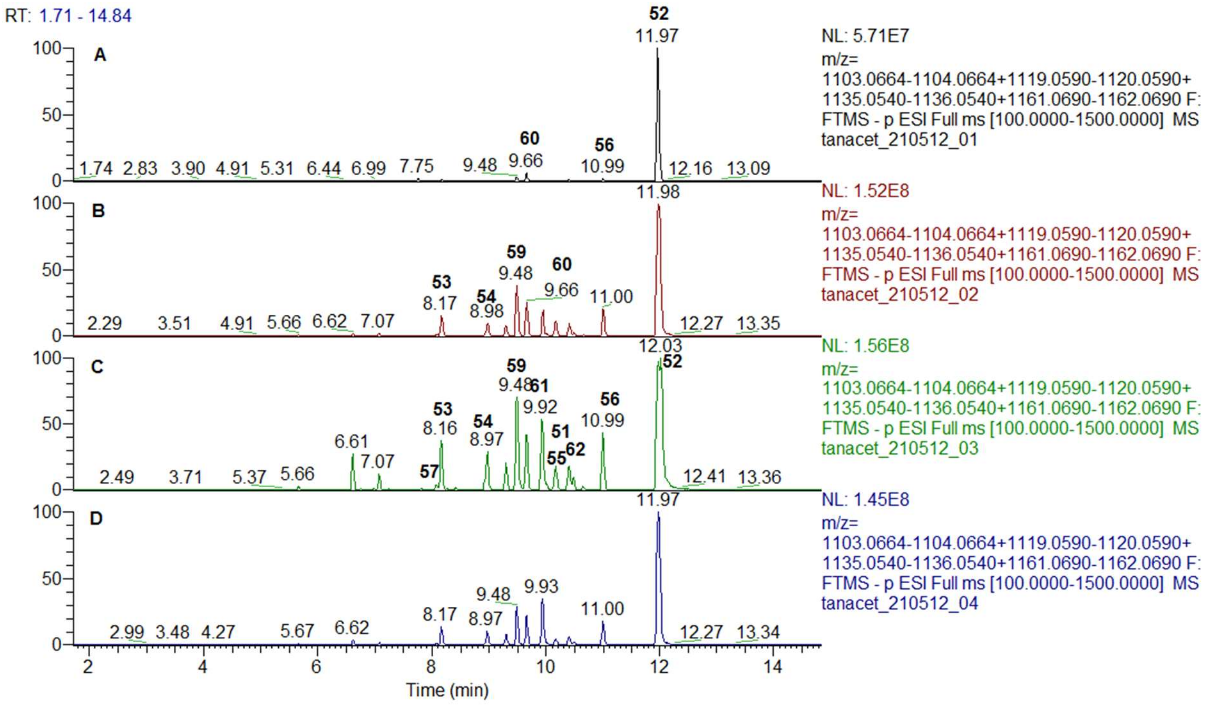

Figure S4. Extracted ion chromatograms of the assayed saponins in *Primula auriculata* aerial parts: ethylacetate extract (A); ethanol extract (B); ethanol/water extract (C); infusion extract (D)

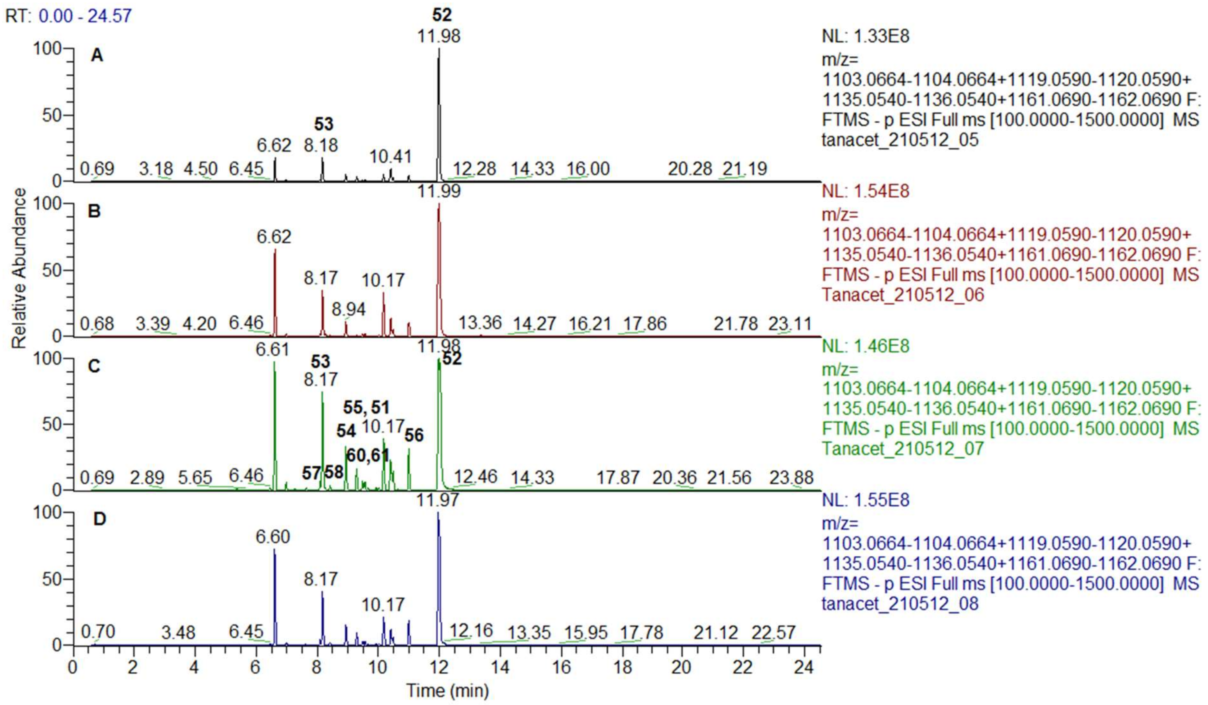

Figure S5. Extracted ion chromatograms of the assayed saponins in *Primula auriculata* roots: ethylacetate extract (A); ethanol extract (B); ethanol/water extract (C); infusion extract (D)
